# Increased Risk of Acute Kidney Injury following Pneumococcal Pneumonia: A Nationwide Cohort Study

**DOI:** 10.1371/journal.pone.0158501

**Published:** 2016-06-30

**Authors:** Te-Yu Lin, Yu-Guang Chen, Cheng-Li Lin, Chia-Hung Kao

**Affiliations:** 1 Division of Infectious Diseases and Tropical Medicine, Department of Internal Medicine, Tri-Service General Hospital, National Defense Medical Center, Taipei, Taiwan; 2 Division of Hematology and Oncology, Department of Internal Medicine, Tri-Service General Hospital, National Defense Medical Center, Taipei, Taiwan; 3 College of Medicine, China Medical University, Taichung, Taiwan; 4 Management Office for Health Data, China Medical University Hospital, Taichung, Taiwan; 5 Department of Nuclear Medicine and PET Center, China Medical University Hospital, Taichung, Taiwan; 6 Graduate Institute of Clinical Medical Science, School of Medicine, College of Medicine, China Medical University, Taichung, Taiwan; University of Sao Paulo Medical School, BRAZIL

## Abstract

**Purpose:**

Pneumococcal disease leads to renal complications ranging from persistent proteinuria to end-stage renal disease. Studies on the association between pneumococcal pneumonia (PP) and acute kidney injury (AKI) are scant. This study assessed the relationship between PP and risk of AKI.

**Methods:**

This nationwide population-based cohort study examined data from the Taiwan National Health Insurance Research Database for the period 2000–2011. We identified inpatients with newly diagnosed PP according to the International Classification of Diseases, Ninth Revision, Clinical Modification (ICD-9-CM) codes. In addition, we selected a comparison cohort from inpatient claims without the diagnosis of PP that was randomly frequency-matched with the PP cohort according to age, sex, index year and comorbidities. We analyzed the risks of AKI by using Cox proportional hazards regression models, adjusted for sex, age, and comorbidities.

**Results:**

A total of 10,069 patients with PP and 10,069 controls were enrolled in this study. After adjustments for age, sex, and comorbidities, patients with PP had a 1.11-fold risk of developing AKI compared with the comparison cohort.

**Conclusion:**

This study indicates that AKI risks are higher in patients with PP compared with the comparison cohort. Careful follow-up observation and aggressive treatment are necessary for patients with PP to reduce the risk of AKI.

## Introduction

Pneumococcal pneumonia (PP) is a common disease worldwide and a major concern because of its high morbidity and mortality rates [1]. This disease can cause septic shock, acute respiratory failure, bacteremia, empyema, and meningitis in its acute stage [2,3]. Severe morbidities after the acute stage have been observed. PP is associated with increased risk of stroke [4], acute cardiac events [5,6], lung cancer [7], and end-stage renal disease [8].

Acute kidney injury (AKI) is a common problem in critically ill patients. AKI occurs in up to 70% of critically ill patients and has a mortality rate in this group more than twice that of similar patients without AKI [9]. Among the traditional causes of AKI, sepsis is the most common etiology [10]. A short episode of AKI may predispose the patient to permanent kidney damage. Coca et al observed an association between AKI and chronic kidney disease [11]. Clinicians should aggressively prevent AKI to avoid adverse outcomes.

PP can cause hemolytic-uremic syndrome in pediatric patients. The disease often results in AKI that requires emergent dialysis [12,13]. Epidemiological studies on the relationship between PP in adults and AKI development are scant. Therefore, we conducted a nationwide population-based cohort study to investigate the association between PP and subsequent risk of AKI.

## Methods

### Data Source

The National Health Insurance (NHI) program was established in Taiwan in March 1995, and currently has more than 23.75 million enrollees, covering more than 99% of the population. The National Health Research Institutes maintain the National Health Insurance Research Database (NHIRD), which contains all NHI claims data. To protect patient privacy, all medical records in the NHIRD are linked through a unique encrypted identifier. In this study, we used the NHIRD inpatient dataset and Registry of Beneficiaries. Diagnoses in the database are based on the International Classification of Diseases, Ninth Revision, Clinical Modification (ICD-9-CM) codes. The Institutional Research Ethic Committee of China Medical University (CMUH104-REC2-115) exempted this study from full review.

### Study Participants

We used inpatient claims data to identify patients aged 20 years or more who were newly diagnosed with PP (ICD-9-CM code 481) between 2000 and 2011. The index date was defined as the date of initial PP diagnosis. We excluded patients with missing sex or date of birth data, and aged <20 years. The comparison cohort was randomly selected from inpatient claims without the diagnosis of PP. For each patient with PP, 1 control patients without PP were randomly selected and frequency-matched according to year of hospitalization, age (every 5 y), sex, and comorbidities of cirrhosis, cancer, chronic kidney disease (CKD), diabetes, hypertension, hyperlipidemia, chronic obstructive pulmonary disease (COPD), congestive heart failure (CHF), coronary artery disease (CAD), and stroke. The same exclusion criteria as used for the PP group were applied for the control group. All the study participants were followed until they were diagnosed with AKI (ICD-9-CM code 584), censored for loss to follow-up, withdrew from the NHI program, or until December 31, 2011.

### Outcome and Comorbidities

In Taiwan, the AKI diagnosis before the year 2004 was based on diagnosis guideline at that time and use RIFLE criteria after the year 2004. These diagnostic codes in NHIRD would be obtained from hospital records and evaluated by two or more specialist to confirm the diagnostic accuracy.

The person-years of the follow-up were calculated for each patient until they were newly diagnosed with AKI, censored for loss to follow-up, withdrew from the insurance program, or until the end of 2011.

Comorbidities, namely cirrhosis (ICD-9-CM code 571.2, 571.5, 571.6), cancer (ICD-9-CM codes 140–208), CKD (ICD-9-CM code 585), diabetes (ICD-9-CM code 250), hypertension (ICD-9-CM codes 401–405), hyperlipidemia (ICD-9-CM code 272), chronic obstructive pulmonary disease (COPD; ICD-9-CM codes 491, 492, and 496), congestive heart failure (CHF; ICD-9-CM code 428), coronary artery disease (CAD; ICD-9-CM codes 410–414), and stroke (ICD-9-CM codes 430–438), were identified according to diagnosis prior to the AKI event. In addition, sepsis (ICD-9-CM codes 0389) was also added in the multivariable analysis. The severity were identified according to hospitalization of PP with continuous mechanical ventilation (high) or without continuous mechanical ventilation (low) (ICD-9 procedure code 967).

### Ethics Statement

The NHIRD encrypts patient personal information to protect privacy and provides researchers with anonymous identification numbers associated with relevant claims information, including sex, date of birth, medical services received, and prescriptions. Therefore, patient consent is not required to access the NHIRD. This study was approved to fulfill the condition for exemption by the Institutional Review Board (IRB) of China Medical University (CMUH104-REC2-115). The IRB also specifically waived the consent requirement.

### Statistical Analysis

The baseline distribution of demographic characteristics and comorbidities were evaluated using the chi-square test for categorical variables and *t* test for continuous variables between the PP and non-PP cohorts. We calculated the overall incidence density rates of AKI and age-, sex-, and comorbidity-specific rates of AKI (per 1000 person-y). Univariate and multivariate Cox proportion hazards regression models were used to examine the influence of PP on the risk of AKI, expressed as a hazard ratio (HR) with a 95% confidence interval (CI). The multivariate models were simultaneously adjusted for sepsis, age, sex, and comorbidities of cirrhosis, cancer, CKD, diabetes, hypertension, hyperlipidemia, COPD, CHF, CAD, and stroke. Stratified by sepsis, age, sex, comorbidity, follow-up time and the severity of PP, the relative risk of AKI development in the patients with PP, compared with the patients without PP, was also analyzed using the Cox models. The Cox model was also used to calculate the adjusted cumulative incidence of AKI for both PP and non-PP cohorts. All statistical analyses were conducted using SAS software Version 9.4 (SAS Institute, Inc., Cary, NC, USA). A 2-tailed *P* value of < .05 was considered statistically significant.

## Results

**Fig 1:**
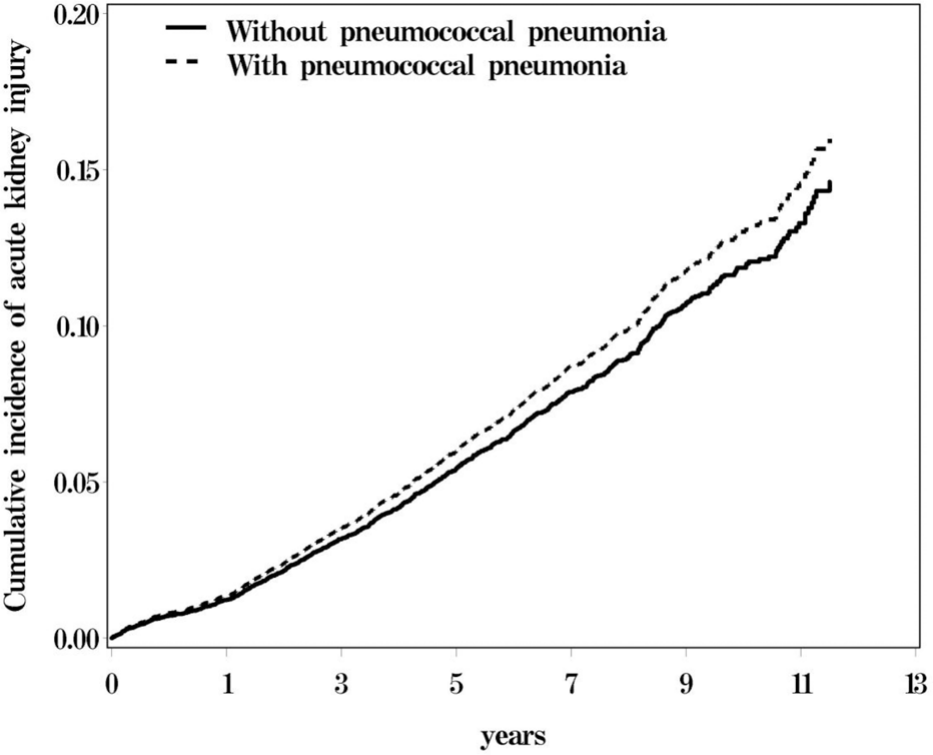
Kaplan-Meier survival analysis showed that the pneumococcal pneumonia group exhibited significantly higher acute kidney injury rates than the comparison group.

The cohort comprised 10 069 PP cases and 10 069 non-PP controls for the period 2000–2011. In both cohorts, approximately 52.5% of the patients were more than 65 years old and 64.1% were men. The mean ages of the PP and non-PP cohorts were 62.7 ±18.5 and 62.2±18.7 years, respectively. The patients with PP and with non-PP had similar prevalence of cirrhosis, cancer, CKD, diabetes, hypertension, hyperlipidemia, COPD, CHF, CAD, and stroke (all *P*>.05) (Table 1). The cumulative incidence of AKI was higher for the PP cohort than for the non-PP cohort (*P* = .006) (Fig 1). In total, 429 patients with AKI were observed in the PP cohort, for an incidence rate of 10.5 per 1000 person-years; 542 patients with AKI were identified in the non-PP cohort, for an incidence rate of 10.5 per 1000 person-years, yielding a crude HR of 1.00 (95% CI = 0.92–1.08) (Table 2). After adjustment for age, sex, and comorbidities, the patients with PP exhibited an increased risk of AKI compared with those without PP (adjusted HR [aHR] = 1.11, 95% CI = 1.03–1.19). The AKI incidence increased with age and was greater in male patients than in female patients. We also observed a significantly higher risk of AKI in the patients with cirrhosis (aHR = 2.58, 95% CI = 2.31–2.89), diabetes (aHR = 1.69, 95% CI = 1.56–1.82), hypertension (aHR = 1.23, 95% CI = 1.13–1.34), CHF (aHR = 1.56, 95% CI = 1.42–1.71), and stroke (aHR = 1.11, 95% CI = 1.03–1.21) than among those with no comorbidity.

**Table 1:** Demographic characteristics and comorbidities in cohorts with and without pneumococcal pneumonia.

	Pneumococcal pneumonia		
	No	Yes	
Variable	N = 10069	N = 10069	*p*-value
**Age, year**			0.99
≤ 49	2627(26.1)	2627(26.1)	
50–64	2160(21.5)	2160(21.5)	
65–79	3381(33.6)	3380(33.6)	
≥80	1901(18.9)	1902(18.9)	
Mean±SD^†^	62.2(18.7)	62.7(18.5)	0.05
**Sex**			0.99
Female	3616(35.9)	3616(35.9)	
Male	6453(64.1)	6453(64.1)	
**Comorbidity**			
Cirrhosis	641(6.37)	641(6.37)	0.99
Cancer	1690(16.8)	1690(16.8)	0.99
CKD	156(1.55)	156(1.55)	0.99
Diabetes	2613(26.0)	2613(26.0)	0.99
Hypertension	4203(41.7)	4203(41.7)	0.99
Hyperlipidemia	875(8.69)	875(8.69)	0.99
COPD	3105(30.8)	3105(30.8)	0.99
CHF	1430(14.2)	1430(14.2)	0.99
CAD	2107(20.9)	2107(20.9)	0.99
Stroke	2266(22.5)	2266(22.5)	0.99

Chi-Square Test

^†^: T-Test

CKD denotes chronic kidney disease

CAD denotes coronary artery disease

CHF denotes congestive heart failure

COPD denotes chronic obstructive pulmonary disease

**Table 2:** Incidence and Hazard ratio for acute kidney injury and acute kidney injury -associated risk factor.

Variable	Event	PY	Rate^#^	Crude HR (95% CI)	Adjusted HR^†^ (95% CI)
**Pneumococcal pneumonia**					
No	542	51380	10.5	1.00	1.00
Yes	429	40748	10.5	1.00(0.92, 1.08)	1.11(1.03,1.19)**
**Comorbidity**					
**Cirrhosis**					
No	848	87139	9.73	1.00	1.00
Yes	123	4990	24.7	2.53(2.26, 2.84)***	2.58(2.31, 2.89)***
**Cancer**					
No	811	80110	10.2	1.00	1.00
Yes	160	12019	13.3	1.32(1.19, 1.46)***	0.94(0.85,1.04)
**CKD**					
No	953	91122	10.5	1.00	1.00
Yes	18	1007	17.9	1.71(1.29, 2.27)***	0.99(0.76, 1.30)
**Diabetes**					
No	554	69904	7.93	1.00	1.00
Yes	417	22224	18.8	2.37(2.19, 2.56)***	1.69(1.56, 1.82)***
**Hypertension**					
No	369	55249	6.68	1.00	1.00
Yes	602	36880	16.3	2.44(2.26, 2.64)***	1.23(1.13,1 .34)***
**Hyperlipidemia**					
No	870	83684	10.4	1.00	1.00
Yes	101	8445	12.0	1.15(1.02, 1.30)***	0.89(0.78, 1.01)
**COPD**					
No	604	64548	9.36	1.00	1.00
Yes	367	27581	13.3	1.42(1.31, 1.54)***	0.94(0.87, 1.02)
**CHF**					
No	703	80151	8.77	1.00	1.00
Yes	268	11978	22.4	2.55(2.34, 2.78)***	1.56(1.42,1 .71)***
**CAD**					
No	654	73201	8.93	1.00	1.00
Yes	317	18928	16.8	1.8791.73, 2.03)***	0.97(0.89, 1.06)
**Stroke**					
No	642	73647	8.72	1.00	1.00
Yes	329	18481	17.8	2.04(1.88, 2.21)***	1.11(1.03, 1.21)*

^#^: incidence rate, per 1,000 person-years

Crude HR, relative hazard ratio

^†^: multivariable analysis including age, sex, and comorbidities of cirrhosis, cancer, CKD, diabetes, hypertension, hyperlipidemia, COPD, CHF, CAD, and stroke

*p<0.05

**p<0.01

***p<0.001.

The risk of AKI in the patients with PP was significantly higher than that in patients without PP for without sepsis group (aHR = 1.09, 95% CI = 1.01–1.18), with sepsis group (aHR = 1.16, 95% CI = 1.06–1.26), aged 65-79y group (aHR = 1.28, 95% CI = 1.13–1.45), and males (aHR = 1.17, 95% CI = 1.07–1.29) (Table 3). The stratified analysis conducted according to the follow-up duration showed that the PP cohort to non-PP cohort developed the highest risk of AKI within 1 year of follow-up (aHR = 1.97, 95% CI = 1.79–2.16). Furthermore, the PP cohort with continuous mechanical ventilation exhibited a significantly much higher risk of AKI compared to the non-PP cohort (aHR = 2.09, 95% CI = 1.81–2.42) (Table 4).

**Table 3:** Incidence of acute kidney injury by age, sex and comorbidity and Cox model measured hazards ratio for patients with pneumococcal pneumonia compared those without pneumococcal pneumonia.

	Pneumococcal pneumonia							
	No			Yes				
Variables	Event	PY	Rate^#^	Event	PY	Rate^#^	Crude HR (95% CI)	Adjusted HR^†^ (95% CI)
**Acute kidney injury without sepsis**	408	51380	7.94	320	40748	7.85	2.07(1.91, 2.25)***	1.09(1.01, 1.18)*
**Acute kidney injury with sepsis**	134	51380	2.61	109	40748	2.67	1.96(1.78, 2.15)***	1.16(1.06, 1.26)**
**Age, years**								
≤ 49	41	15827	2.59	38	14585	2.61	1.01(0.85, 1.20)	1.10(0.94, 1.29)
50–64	86	11509	7.47	76	9612	7.91	1.06(0.89, 1.26)	1.09(0.92, 1.29)
65–79	236	17147	13.8	216	12331	17.5	1.27(1.12, 1.44)***	1.28(1.13, 1.45)***
≥80	179	6897	26.0	99	4220	23.5	0.90(0.77, 1.06)	0.90(0.76, 1.06)
**Sex**								
Female	180	19466	9.25	127	16319	7.78	0.84(0.74, 0.96)*	0.98(0.87, 1.11)
Male	362	31914	11.3	302	24429	12.4	1.09(0.99, 1.20)	1.17(1.07, 1.29)***
**Comorbidity**^**‡**^								
No	33	15714	2.10	20	14454	1.38	0.66(0.38, 1.15)	0.77(0.44, 1.36)
Yes	509	35667	14.3	409	26294	15.6	1.09(0.95, 1.24)	1.14(1.00, 1.30)
**Follow-up time, years**								
≤1	85	9460	8.98	139	8224	16.9	1.88(1.71, 2.07)***	1.97(1.79, 2.16)***
2–4	215	21421	10.0	146	16815	8.68	0.87(0.70, 1.07)	0.96(0.78, 1.19)
≥5	242	20499	11.8	144	15709	9.17	0.78(0.63, 0.96)*	0.94(0.76, 1.15)

^#^: incidence rate, per 1,000 person-years

Crude HR, relative hazard ratio

^†^: multivariable analysis including sepsis, age, sex, and comorbidities of diabetes, hypertension, hyperlipidemia, COPD, CHF, CAD, stroke, cirrhosis, cancer, and CKD

^‡^: Patients with any one of the comorbidities diabetes, hypertension, hyperlipidemia, COPD, CHF, CAD, stroke, cirrhosis, cancer, and CKD as the comorbidity group

*p<0.05

**p<0.01

***p<0.001.

**Table 4:** Cox Proportional Hazard Regression Analysis for the risk of acute kidney injury stratified by the severity of pneumococcal pneumonia.

Variables	N	Event	Rate^#^	Adjusted HR^†^ (95% CI)
**Non-pneumococcal pneumonia**	10069	542	10.6	1(Reference)
**Pneumococcal pneumonia severity**^**&**^				
Without continuous mechanical ventilation(Low)	8650	362	9.46	1.02(0.94, 1.10)
With continuous mechanical ventilation(High)	1419	67	27.1	2.09(1.81, 2.42)***

^#^: incidence rate, per 1,000 person-years

^†^: multivariable analysis including age, sex, and comorbidities of diabetes, hypertension, hyperlipidemia, COPD, CHF, CAD, stroke, cirrhosis, cancer, and CKD

***P< .001

^&^severity were identified according to hospitalization of pneumococcal pneumonia with continuous mechanical ventilation (High) or without continuous mechanical ventilation (Low).

## Discussion

AKI is an abrupt loss of renal function within a short time; sepsis is the most common etiology of the condition. *Streptococcus pneumonia* is a common etiology of pneumonia with sepsis and, in its acute stage, can increase the risk of AKI. Sepsis-mediated hypoperfusion and hypoxemia may result in peritubular hypoxia and then cause AKI [14,15]. We observed the highest risk of AKI during the first follow-up year after PP. The stratified analysis conducted according to with or without sepsis showed that the PP cohort developed higher risk of AKI compared to non-PP cohort. This phenomenon may be explained by the growing evidence of cellular and inflammatory-mediated injury observed in addition to hypoperfusion in sepsis-associated AKI pathophysiology [16–19]. The surface capsular polysaccharides of pneumococcus trigger host inflammatory responses and induce the production of cytokines [20]. Panichi et al reported that increased levels of inflammation markers such as C-reactive protein and interleukin-6 are predictors of deteriorating renal function in elderly patients [21]. Ficek et al showed that tumor necrosis factor-α is a contributing factor for acute renal failure in sepsis [22] and induces renal interstitial fribrosis by increasing production of transforming growth factor-β1 [23]. Wittenhagen et al found that an increased soluble urokinase-type plasminogen activator receptor (suPAR) level reflects an increased expression of inflammatory cells in vessels during pneumococcal sepsis [24]. An elevated suPAR level during PP might reflect ongoing inflammation that contributes to subsequent podocyte damage, thus resulting in AKI. The findings of these studies are consistent with our epidemiological results.

In this study, the comorbidities and coexistent conditions associated with the development of AKI between the study cohort and comparison cohort were similar. PP remained an independent risk factor for developing AKI after covariates were adjusted. The AKI incidence increased with age in this study was consistent with previous report. Kidney function deteriorates with age, thus increasing AKI risk [25].

Our multivariable regression analysis revealed elevated AKI rates among the patients with diabetes, hypertension, congestive heart failure, cirrhosis and stroke. These results are consistent with previous studies. Furthermore, the HR of AKI was higher in the younger patients with PP than in the older ones; this is possibly because the older subgroup exhibited more comorbidities and underlying medical illnesses, which may have reduced the effect of PP on their AKI. This phenomenon was also observed in the comorbidity-stratified results.

This study has several limitations. First, the NHIRD does not contain detailed information about the current medications of the patient or hydration fluid status which are potential confounding factors that might have influenced the study outcomes. Second, the insurance claims database do not contain detailed information regarding patients with PP, including clinical factors related to the severity of the infection, such as shock, laboratory values of white blood cell count, platelet count, blood urea nitrogen and serum creatinine levels (AKI severity), all of which might be confounding factors in this study. However, we use indicator ''continuous mechanical ventilation'' as severity of PP. Third, the evidence derived from a retrospective cohort study is generally lower in statistical quality than that from randomized trials because of potential biases related to adjustments for confounding variables. Fourth, a prospective patient registration survey is required to monitor the change of estimated glomerular filtration rate in patients after PP infection.

In conclusion, the patients hospitalized for PP exhibited an increased risk of AKI compared with inpatients without PP. One episode of PP might exert clinically substantial renal effects. We recommend that physicians carefully monitor renal function when treating patients with a history of PP.

## Abbreviations

PP: pneumococcal pneumonia
AKI: acute kidney injury
aHR: adjusted hazard ratio
CI: confidence interval
NHIRD: National Health Insurance Research Database
BNHI: Bureau of National Health Insurance
NHI: National Health Insurance
NHIA: National Health Insurance Administration
NHRI: National Health Research Institutes
LHID 2000: Longitudinal Health Insurance Database 2000
